# The impact of human and mouse differences in NOS2 gene expression on the brain’s redox and immune environment

**DOI:** 10.1186/1750-1326-9-50

**Published:** 2014-11-17

**Authors:** Michael D Hoos, Michael P Vitek, Lisa A Ridnour, Joan Wilson, Marilyn Jansen, Angela Everhart, David A Wink, Carol A Colton

**Affiliations:** Department of Neurosurgery, Stonybrook Health Sciences, Stony Brook, NY 11794 USA; Department of Neurology, Duke University Medical Center, Durham, NC 27710 USA; Radiation Biology Branch, National Cancer Institute, National Institutes of Health, Bethesda, MD 20892 USA

**Keywords:** NOS2, Mouse models, Neurodegeneration, Redox, Inflammation, Nitric oxide

## Abstract

**Background:**

Mouse models are used in the study of human disease. Despite well-known homologies, the difference in immune response between mice and humans impacts the application of data derived from mice to human disease outcomes. Nitric oxide synthase-2 (*NOS2*) is a key gene that displays species-specific outcomes via altered regulation of the gene promoter and via post-transcriptional mechanisms in humans that are not found in mice. The resulting levels of NO produced by activation of human *NOS2* are different from the levels of NO produced by mouse *Nos2*. Since both tissue redox environment and immune responsiveness are regulated by the level of NO and its interactions, we investigated the significance of mouse and human differences on brain oxidative stress and on immune activation in *HuNOS2*^*tg*^*/mNos2*^*-/-*^ mice that express the entire human *NOS2* gene and that lack a functional *mNos2* compared to wild type (WT) mice that express normal *mNos2*.

**Methods/results:**

Similarly to human, brain tissue from *HuNOS2*^*tg*^*/mNos2*^*-/-*^ mice showed the presence of a *NOS2* gene 3′UTR binding site. We also identified miRNA-939, the binding partner for this site, in mouse brain lysates and further demonstrated reduced levels of nitric oxide (NO) typical of the human immune response on injection with lipopolysaccharide (LPS). *HuNOS2*^*tg*^*/mNos2*^*-/-*^ brain samples were probed for characteristic differences in redox and immune gene profiles compared to WT mice using gene arrays. Selected genes were also compared against *mNos2*^*-/-*^ brain lysates. Reconstitution of the human *NOS2* gene significantly altered genes that encode multiple anti-oxidant proteins, oxidases, DNA repair, mitochondrial proteins and redox regulated immune proteins. Expression levels of typical pro-inflammatory, anti-inflammatory and chemokine genes were not significantly different with the exception of increased TNFα and *Ccr1* mRNA expression in the *HuNOS2*^*tg*^*/mNos2*^*-/-*^ mice compared to WT or *mNos2*^*-/-*^ mice.

**Conclusions:**

NO is a principle factor in establishing the tissue redox environment and changes in NO levels impact oxidative stress and immunity, both of which are primary characteristics of neurodegenerative diseases. The *HuNOS2*^*tg*^*/mNos2*^*-/-*^ mice provide a potentially useful mechanism to address critical species- specific immune differences that can impact the study of human diseases.

## Background

The past five years have proven to be disappointing and frustrating to pre-clinical scientists in the Alzheimer’s field. Multiple therapeutics that worked to reduce amyloid mediated pathology in established mouse models of Alzheimer’s disease (AD) have not proven to be useful in humans with AD and, in some cases, have made the disease outcomes worse [1–4]. The results of these and other clinical trials have now driven the search for a better understanding of AD disease mechanisms and emphasize the pressing need for re-evaluation of research directions in the field. Recently, a critical avenue of research relevant to neurodegeneration has been a subject of renewed interest. Although immune changes in AD have been known for many years, the importance of an immune response to the disease process was unclear. This was despite convincing evidence from Caleb Finch [5], W. Sue Griffin [6, 7], Piet Eikenlenboom [8], Patrick and Edith McGeer [9, 10] and others who showed that immune responses were observed as an early and key pathological characteristic of humans with AD. Data from genome-wide association studies (GWAS) have now firmly established that genes involved in the immune response are central to the risk for development of late onset Alzheimer’s disease [11–13]. These genes encode proteins that include apolipoprotein E (APOE) that serves as a global immune regulator [14], complement receptor 1(CR1) that may mediate Aβ clearance from the brain [15], CD33 (Siglec11) that when expressed on microglia becomes neuroprotective [16] and TREM2 with its downstream target TYROBP (DAP12) that regulates multiple microglial functional pathways [17, 18]. The immune response in AD is clearly a critical event in the disease process. Zhang et al. [17] using an integrated network analysis of late onset AD-associated genes showed that immune/microglia gene networks demonstrated the strongest degree of disease association and the highest correlation to AD neuropathology compared to other gene subsets. Despite these exciting findings, we still don’t understand how immune cells such as microglia are involved in the initiation or progression of disease, if and when their involvement changes with time or even what type of response is generated by microglia (or other immune cells) at different stages of AD.

Seok et al. [19] have re-enforced an additional dimension of complexity that impacts our methodologies we use to understand how the brain’s immune response is involved in neurodegenerative diseases such as AD. In their controversial study, Seok and colleagues compared the genomic responses of mice and humans during an acute immune response resulting from endotoxemia, burns or trauma. Their study demonstrated that correlations between mouse and human immune signaling pathways were poor for these acute illnesses and at least for pathways that are commonly initiated by lipopolysaccharide (LPS), mouse gene changes were not predictive of human gene changes for a similar disease initiation process. More recently, this interpretation has been challenged by Takao and Miyakawa [20]. Regardless, it is generally agreed that not only are disease-based changes in immunity a challenging issue, but also the models used to study human immune responses during disease should be carefully considered.

The recognition that mouse immune processes are not the same as human immune processes is clearly not new. Mestas and Hughes in 2004 [21] defined a number of differences in both innate and adaptive immune characteristics between rodents and man. These differences include Fc receptor subtypes, the action of IFNα on Th1 cells and the involvement of the Th2 response in clearance of parasites. In 1995 our lab showed that cultured microglia and macrophages from humans showed limited nitric oxide (NO) production in response to immune stimuli that were commonly used on mouse cells in culture such as LPS or polyinosinic:polycytidylic acid (PIC) alone or in combination with cytokines such as interferon- γ (IFNγ) or interleukin-1β [22, 23]. Microglia cultured from rodent brains, however, are well known to produce robust levels of NO when stimulated with the same induction agents. NO is the product of the inducible iNOS protein that is encoded by the *NOS2* gene and serves defensive and regulatory roles in an immune response. Multiple other labs have observed similar differences between NO production in mouse and in human cells and a vigorous discussion on the molecular mechanisms underlying these differences has ensued over the intervening years [24–28]. An elegant study by Guo et al. [29] demonstrated a reasonable explanation for these differences. Guo, Geller and colleagues found binding sites for a microRNA (miRNA-939) in the 3′untranslated region (UTR) of the human *NOS2* gene. When these sites are bound to miRNA-939, a post-transcriptional repression of iNOS protein expression is initiated and NO production was thereby reduced. While this may not be the sole mechanism for differences between human and rodent NO production and differences in promoter regulatory activity also exist [25, 30–32], miRNA-mediated silencing may account for a large part of the inability to readily stimulate and measure NO in human macrophages and microglia.

Despite problems with species-specific responses, it is equally clear that mouse models of disease facilitate the mechanistic study of cellular and integrated systems at multiple stages of a disease process. Animal models will remain a valuable tool to understand human disease. However, the translatability of these data derived from mice to human is ultimately a question. Furthermore, the lack of models that more closely resemble the human immune environment remains a core problem. In addition to differences in immune regulatory events [19, 33, 34], these problems include our lack of knowledge on regulation of the cell and tissue redox environment that also impacts immunity [34, 35].

One way to address these issues is to “humanize” mouse models by replacing mouse genes with corresponding human genes. There are currently many examples of this type of approach including multiple models of neurodegenerative disease where a mutated gene is added in addition to the mouse gene (for example, the APPsw-Tg2576 mice; [36] or where the human gene replaces a large part of the mouse gene with or without replacing the promoter region of the gene (*Apoe4* targeted replacement mice, [37]). Similar methodologies have been used for immune genes. The choice of which mouse gene to alter depends on the disease process.

We have developed mouse models of AD that express mutated human amyloid precursor protein (*APP*) and also demonstrate human-like NO production when immune stimulated. Our scientific rationale for this model was based on the interrelationship between inflammation, oxidative stress and neurodegeneration [34, 38]. NO serves as a nodal point between inflammation and oxidative stress, and when altered, results not only in a changed brain redox environment [34, 35], but also in altered immune regulation, both of which impact human disease [33, 34]. The types of interactions and their products are now well described in excellent reviews and will not be discussed here [39–43]. However, it is clear that both superoxide anion through oxidative events and NO through both direct and indirect reactions regulate multiple proteins and cellular pathways in a precise manner. These pathways dictate immune regulation and immune defense [34]. Because the direct and indirect reactions of NO are dependent on the available concentration of NO, the consumption of NO, for example by combination with superoxide anion, significantly impacts the redox environment of the tissue and alters multiple NO-dependent pathways [42–44]. By shifting the inherent NO level during an immune response, the outcome is likely to provide a unique subset of regulatory and defensive events that are tailored to the individual species. Evolution has made this shift for humans and rodents and has imposed a difference in NOS2 regulation between these species.

To better understand the importance of mouse and human *Nos2* differences, we have generated a mouse model that expresses the entire human *NOS2* gene on a mouse *Nos2* knockout background [45]. This new mouse strain has gene regulatory sites that, similar to the human NOS2 gene, are associated with reduced NO production. Here, we show characteristic redox gene differences between mice expressing a normal *mNos2* gene and mice expressing only the *huNOS2* gene. We also discuss the potential impact of these gene differences on tissue redox balance and immunity.

## Results

### *HuNOS2*^*tg*^*/mNos2*^*-/-*^mice show 3′UTR binding sites, miRNA-939 homolog and reduced NO production *in vivo*

**Figure 1:**
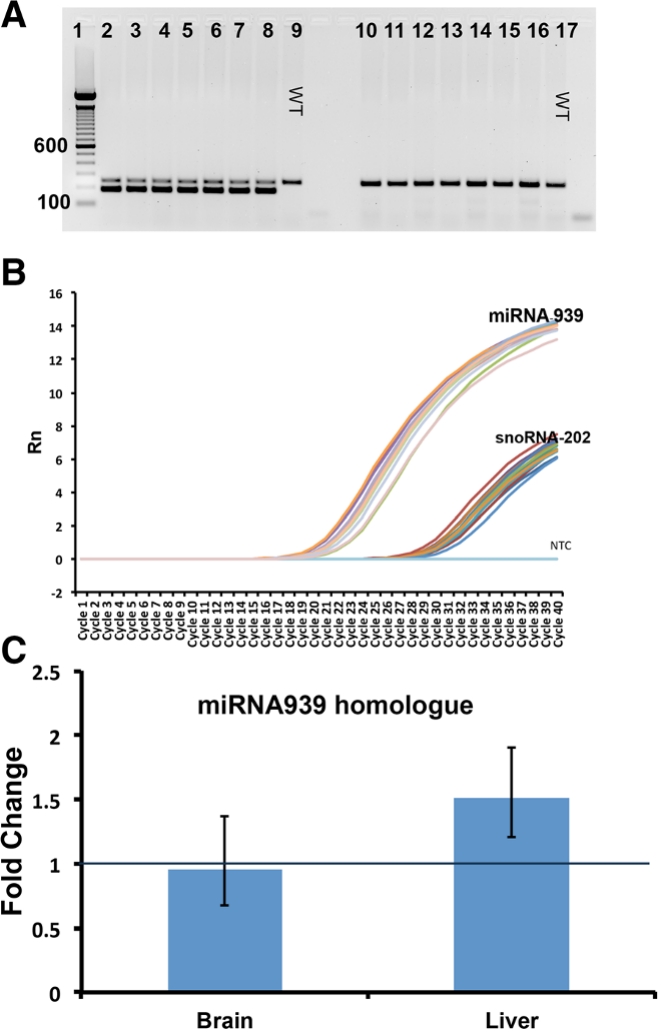
***HuNOS2*** ^***tg***^ ***/mNos2*** ^***-/-***^ **mice express both the 3′UTR binding site and a miRNA-939 homolog involved in regulation of the** ***huNOS2*** **gene. A**- PCR amplification of the 3′UTR region containing putative binding sites for miRNA-939 of the human NOS2 gene in *HuNOS2* ^*tg*^ */mNos2* ^*-/-*^ and WT mice. Lanes represent individual mice. Lower bands in Lanes 2–8 are consistent with the human 3′UTR region which is not observed in WT mice (lanes 9–17). **B**- Graph of PCR product amount versus cycle number for amplification of miRNA-939 homolog product using small nucleolar (sno) RNA-202 as endogenous controls. **C**- Relative change in miRNA-939 homolog levels in brain and liver lysates at 7 hrs after injection of LPS compared to saline injected lysates. Data points represent average fold change (±sem), n = 3 mice each strain.

Guo et al. [31] have shown that miRNA-939 binds to sites in the 3′UTR region of the human *NOS2* gene, thereby altering the translation of iNOS protein and reducing NO production. To determine if the human NOS2 gene in *HuNOS2*^*tg*^*/mNos2*^*-/-*^ mice expressed the appropriate 3′UTR binding sites we PCR amplified the miRNA-939 binding region from DNA isolated from *HuNOS2*^*tg*^*/mNos2*^*-/-*^ brain tissue. Figure 1A shows the results of a typical genotyping assay for 7 individual *HuNOS2*^*tg*^*/mNos2*^*-/-*^ mice and corresponding WT mice. The upper band found in mice expressing the *HuNOS2* gene corresponds to a region of the *mNos2* gene, which has remained intact in the *mNos2*^*-/-*^ strain despite the disruption to the *mNos2* gene. The lower band corresponds to a 185 bp product containing the 3′UTR miRNA-939 binding region of *huNOS2* gene.

Having demonstrated that *HuNOS2*^*tg*^*/mNos2*^*-/-*^ mice express the 3′UTR binding site that is characteristic of the human *NOS2* gene, we then used RT-PCR to detect the presence of a miRNA-939 homolog in brain and liver lysates from lipopolysaccharide (LPS)- treated *HuNOS2*^*tg*^*/mNos2*^*-/-*^ mice. LPS diluted into saline was injected intravenously (*iv)* to induce an immune response that includes induction of the NOS2 gene and immune-related cytokines. Saline-injected mice were used as untreated controls. At 7 hrs after injection, we assayed brain samples for miRNA-939 and used small nucleolar RNA-202 (snoRNA-202) as an endogenous control for microRNAs. Figure 1B presents results from a typical PCR cycling reaction and demonstrates the presence of miRNA-939 in *HuNOS2*^*tg*^*/mNos2*^*-/-*^ brain. We also examined the effect of LPS treatment on the level of miRNA-939 homolog in our mice. Figure 1C shows the average fold change (±sem) in miRNA-939 in brain and liver samples after stimulation with LPS. A similar LPS-mediated increase in miRNA939 levels was shown by Guo et al. [29] for human hepatocytes in culture and in liver lysates from WT mice injected with LPS or a cytokine mix.

**Figure 2:**
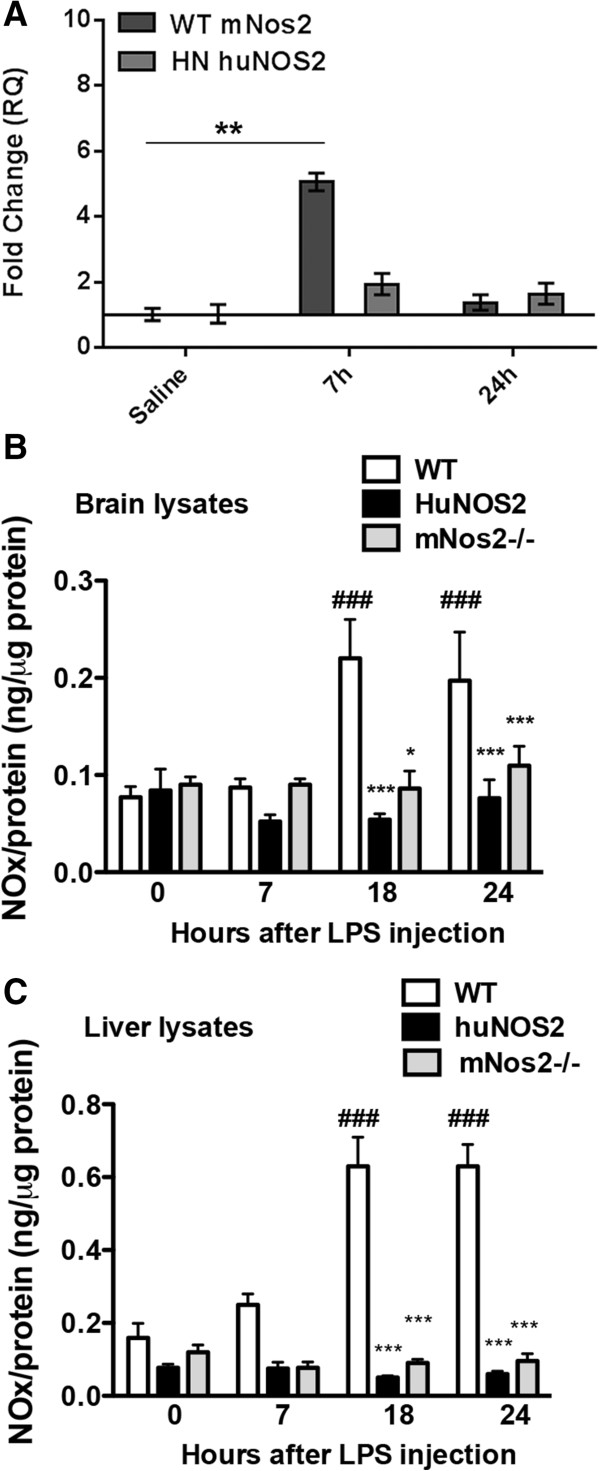
**LPS-stimulated mRNA and NOx production in** ***HuNOS2*** ^***tg***^ ***/mNos2*** ^***-/-***^ **mice** ***.*** **A**. Comparison of the fold changes in NOS2 mRNA levels for *HuNOS2* and *mNos2 (WT)* mice. Brain lysates were prepared from saline injected and LPS injected WT and *HuNOS2* ^*tg*^ */mNos2* ^*-/-*^ mice at 7 and 24 hrs. Average fold changes (±sem) for LPS-treatment were determined using the average saline-injected value as comparator. n = 3 mice/group. ** = p <0.01 using the unpaired student’s *t* test with significance set at p < .0.05. **B**. Brain lysate levels of nitrite and nitrate (NOx) were measured in *HuNOS2* ^*tg*^ */mNos2* ^*-/-*^, *mNos2* ^*-/-*^ and WT mice at 7, 18 and 24 hrs. Saline- injected mice from each strain served as the 0 hrs time point. Average NOx values (ng/ug protein ± sem) are shown where n = 6-9 mice/strain per time point. Significance with time within strain was determined using one-way ANOVA while statistical significance across genotypes was determined using two-way ANOVA (GraphPad Software, San Diego CA). Significance was set at p ≤0.05. *p <0.05; ***p <0.001 for comparisons across strains; ###p <0.001 for comparisons within strain. **C**. Analysis of NOx in liver lysates from the same mice as above and under the same conditions. ***p <0.001 for comparisons across strains; ###p <0.001 for comparisons within strain.

Our previously published studies using LPS or cytokine-stimulated primary cultures of peritoneal macrophages derived from *HuNOS2*^*tg*^*/mNos2*^*-/-*^ mice demonstrated a significant decrease in the conversion of [H^3^] arginine to [H^3^] citrulline [45], indicating reduced iNOS enzymatic activity compared to WT mice. Human and mouse NOS2 have similar specific activity suggesting that changed activity is regulated via cellular processes [46]. In addition, we showed that NO production in immune stimulated cultured peritoneal macrophages was also significantly decreased in *HuNOS2*^*tg*^*/mNos*^*-/-*^ mice compared to similarly treated WT mice. To show that *in vivo* NO production was also altered we measured total nitrite plus nitrate (NOx) levels in whole brain lysates in *HuNOS2*^*tg*^*/mNos2*^*-/-*^ mice compared to similarly treated *mNos2*^*-/-*^ and WT mice. All mice (36–40 weeks of age) were intravenously (iv) injected with either 10 mg/kg of LPS or saline (0.9% NaCl) via the tail vein and tissues were collected at either 7,18 or 24 hrs post injection. Figure 2A show mRNA levels for LPS-treated mice compared to untreated (saline-injected) conditions for both WT and *HuNOS2*^*tg*^*/mNos2*^*-/-*^ mice brain lysates. As predicted, mRNA increased in both strains on LPS stimulation, however, levels of expression were lower in mice expressing the *huNOS2* gene. We next compared nitrite and nitrate (NOx) levels in brain (Figure 2B) and liver (Figure 2C) lysates derived from LPS-injected or saline-injected WT mice to the levels of NOx found in lysates from *mNos2*^*-/-*^ and *HuNOS2*^*tg*^*/mNos2*^*-/-*^ mice. As predicted, WT mice demonstrated a significantly increased level of NOx in both brain and liver tissue lysates with time after immune stimulation. However, neither *mNos2*^*-/-*^ knockout mice nor *HuNOS2*^*tg*^*/mNos2*^*-/-*^ mice showed a measurable *in vivo* response to treatment with LPS when compared to WT-*Nos2* sufficient mice.

### *HuNOS2*^*tg*^*/mNos2*^*-/-*^mice show a unique redox gene profile

**Figure 3:**
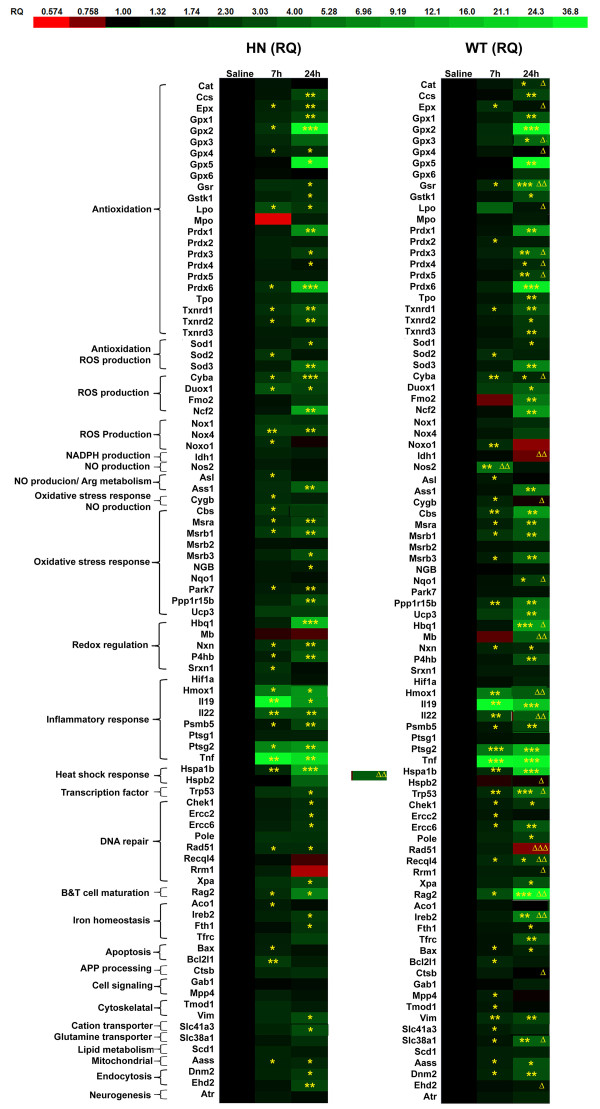
**Comparison of redox gene expression levels between LPS-simulated** ***HuNOS2*** ^***tg***^ ***/mNos2*** ^***-/-***^ **mice and WT mice.** *HuNOS2* ^*tg*^ */mNos2* ^*-/-*^ and WT mice were injected with 10 mg/kg LPS, or with saline as a control, and brains were removed after 7 or 24 hours. RNA was extracted, reverse transcribed, and target gene expression was measured by q-PCR. Fold changes (RQ) were determined for *HuNOS2* ^*tg*^ */mNos2* ^*-/-*^ and WT mice using appropriate saline-injected animals as the comparator for each mouse strain. RQ values were linearized by Log2 conversion, and then subjected to conditional formatting whereby red cells denote lower expression and green cells denote higher expression according to the color scale. Significant differences in gene expression for LPS treated as compared to same strain saline-injected controls were determined by student’s *t*-test (*p ≤0.05, **p <0.01, ***p <0.001). Significant differences in gene expression between WT and *HuNOS2* ^*tg*^ */mNos2* ^*-/-*^ mice at specific time points were determined by student’s *t*-test (Δ p ≤0.05, ΔΔ p <0.01, ΔΔΔ p <0.001). n = 3 mice per group.

To better understand how reconstitution of the human *NOS2* gene in a *mNos2*^*-/-*^ knockout background impacts the redox and immune profile compared to WT mice that normally express *mNos2* and high levels of NO, we performed a directed gene array analysis. For this experiment RNA was extracted from brain lysates previously prepared from the WT and *HuNOS2*^*tg*^*/mNos2*^*-/-*^ mice injected with either LPS (10 mg/kg) or saline. Quantitative RT-PCR was performed on the cDNA using a custom TaqMan Gene Expression Assay plate (Life Technologies). The array was designed to evaluate a defined mixture of redox and immune genes and three mice for each treatment group at each time point were assessed. All CT values were normalized to endogenous levels of 18sRNA and average fold change values (RQ) were determined by 2^(-ΔΔCt)^ method using either WT saline- injected or *HuNOS2*^*tg*^*/mNos2*^*-/-*^ saline- injected as the comparator [47]. The resultant RQ values for LPS-treated *HuNOS2*^*tg*^*/mNos2*^*-/-*^ mice (here abbreviated HN) and WT mice samples were then compared at 7 and 24 hrs post-LPS injection. To better visualize the changes due to LPS treatment for each mouse strain and at each time point, a heat map was prepared using these ratios (Figure 3). Ratio values were linearized by Log2 conversion, then subjected to conditional formatting whereby Log2(RQ) values less than 0 were colored red (decreased expression) and Log2(RQ) values greater than 0 were colored green (increased expression) according to the scale in Figure 3. Asterisks denote a statistically significant change due to LPS treatment with respect to control (saline-injected) mice. In addition, we compared the fold-change in expression levels in LPS treated *HuNOS2*^*tg*^*/mNos2*^*-/-*^ mice for each gene to the corresponding fold-change found in WT mice. Statistically significant changes for these data are shown only for the 24 hrs treatment point and are labeled on the heat map with a delta (Δ) sign. Genes are also grouped according to their known function.

LPS stimulation differentially altered the expression of redox related genes in both a time and gene specific manner in the *HuNOS2*^*tg*^*/mNos2*^*-/-*^ mice compared to WT mice. For example, as shown in the heat map, DNA repair genes that are critical to reducing oxidative DNA damage such as Excision Repair Cross Complementation 2 (*Ercc2*) that encodes the XPD protein and Ercc6 that encodes the Cockayne syndrome b (CSB) protein show no significant gene induction in *HuNOS2*^*tg*^*/mNos2*^*-/-*^ mice at 7 hrs post-LPS injection. By 24 hrs, however, expression of these genes in response to LPS-injection in *HuNOS2*^*tg*^*/mNos2*^*-/-*^ mice was observed, indicating a different time to response in *HuNOS2*^*tg*^*/mNos2*^*-/-*^ mice vs WT mice. Checkpoint homolog 1 (Chek1) and the transcription factor p53 follow a similar pattern. The significance of this delayed repair response to LPS in *HuNOS2*^*tg*^*/mNos2*^*-/-*^ mice is not clear, but it suggests that NO levels affect the early phases of the innate immune process in a species-specific manner. Similarly, we find that anti-oxidant and oxidant genes show a significantly higher or lower expression level in LPS-treated *HuNOS2*^*tg*^*/mNos2*^*-/-*^ mice at 24 hrs post-injection compared to similarly treated WT mice. Selected genes are also shown in Table 1. As predicted from the role of NO as a regulator of oxidant/antioxidant balance, *HuNOS2*^*tg*^*/mNos2*^*-/-*^ mice show decreased expression (WT > HN) of many antioxidant genes (peroxiredoxins, glutathione reductase (*Gsr*), catalase (*Cat*)) concomitant with increased expression of genes (HN > WT) associated with oxidative pathways such as *Cyba* (the b subunit of the NADPH oxidase), *Lpo* (lactoperoxidase) and eosinophil peroxidase (*Epx*) compared to WT mice. This altered expression pattern is indicative of a shift of the response at 24 hrs potentially to a more “oxidative” environment that is regulated by NO flux. Again, how this exactly impacts the disease process remains unknown but it is likely to dictate a different overall redox microenvironment of affected cells.

**Table 1:** **LPS-stimulated gene expression differences between** ***HuNOS2*** ^***-/-***^ ***/mNos2*** ^***-/-***^ **and WT mice brain at 24 hrs post injection**

Gene abbreviation	Gene name	Significantly different
*Prdx3	Periredoxin 3	WT>HN
Prdx4	Periredoxin 4	WT>HN
Prdx5	Periredoxin 5	WT>HN
Gsr	Glutathione reductase	WT>HN
Cyba	NADPH oxidase Cytochrome- b subunit a	HN>WT
Cat	Catalase	WT>HN
Epx	Eosinophil peroxidase	HN>WT
Gpx4	Glutathione peroxidase 4	HN>WT
Nqo1	NAD(P)H dehydrogenase 1-Quinone 1	WT>HN
Gsr	Glutathione reductase	WT>HN
*Lpo	lactoperoxidase	HN>WT
Ireb2	Iron responsive element binding protein 2	WT>HN
RAD51	RAD51 recombinase	HN>WT
*Recql4	REC-q like protein 4 (helicase)	ND
Hbq1	Hemoglobin theta 1	WT>HN
*IL-22	Interleukin 22	HN>WT
Rag2	Recombination activating gene 2	WT>HN
Slc38a1	Solute Carrier 38a1 (glutamine transporter)	WT>HN

**Figure 4:**
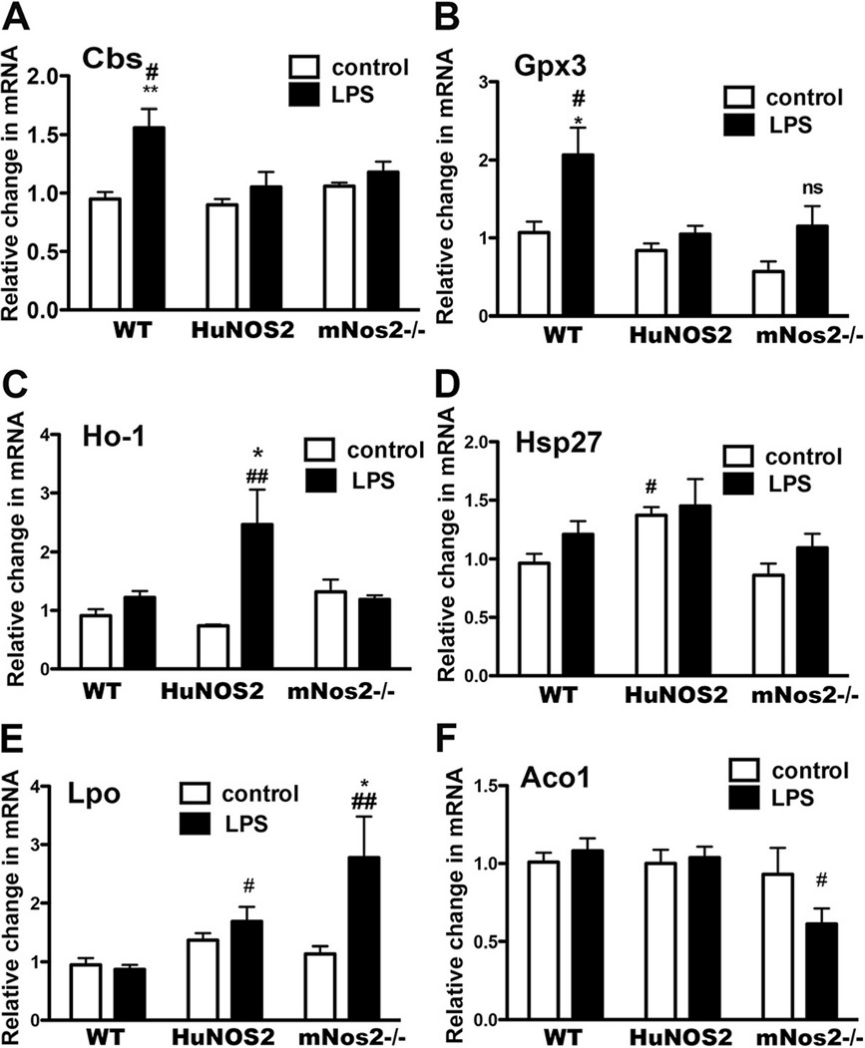
**Comparison of gene expression for selected genes.** Genes that showed changes in expression levels between WT and *HuNOS2* ^*tg*^ */mNos2* ^*-/-*^ mice were also examined for expression levels in *mNos2* ^*-/-*^ mice. Brain lysates were prepared from saline-injected (white bars) and LPS-injected (black bars) *mNos2* knockout mice as described and gene expression measured using qRT-PCR. Average fold changes (±sem) for each gene in saline and LPS-treated conditions were determined using the average WT saline-injected value as comparator. Statistical significance across genotypes and treatment was determined using two-way ANOVA (GraphPad Software, San Diego CA). Significance was set at p ≤0.05. *p <0.05; **p <0.01 for comparisons with treatment; #p <0.05, ##p <0.01 for comparisons across strains. n = 5–6 mice per group.

*Gene analysis was confirmed by additional RT-PCR (See Figure 4).

To better understand how varying NO levels *in vivo* might alter LPS-stimulated gene expression, we further analyzed expression of selected genes in *mNOS2*^*-/-*^ mice (no NO) and compared these mRNA levels to the levels found in high NO (WT mice) and to mice with “human”-like levels (*HuNOS2*^*tg*^*/mNos2*^*-/-*^). Examples of gene expression patterns are shown in Figure 4. We found LPS-stimulated genes (cystathione-b- synthase; glutathione peroxidase-3; Figure 4 A, B) that were significantly changed only in WT mice compared to either *HuNOS2*^*tg*^*/mNos2*^*-/-*^ or *mNOS2*^*-/-*^, and thus appeared to be dependent on high levels of NO production. Alternatively, heme oxygenase -1 (*Ho-1*) and heat shock protein 27 (Hspb2, Hsp27) were specifically altered in the *HuNOS2*^*tg*^*/mNos2*^*-/-*^ mice brain (Figure 4 C, D). *Ho-1* mRNA levels increased with LPS treatment whereas no significant change in *Ho-1* was found for either WT or the *mNos2* knockout mice. Basal expression levels of *Hsp-27*, however, were increased in mice expressing human NOS2 but not in WT or *mNos2*^*-/-*^ mice. Finally, as shown in Figure 4 (E,F) mice lacking *mNos2* and thus unable to increase NO in response to LPS showed significantly higher levels of lactoperoxidase (*Lpo*) and lower levels of aconitase1 (*Aco1*).

Gene changes between *HuNOS2*^*tg*^*/mNos2*^*-/-*^ and WT mice under non- immune stimulated conditions provide insights into the potential physiological impact of an inherent background difference in WT mice that have high levels of NO and in *HuNOS2*^*tg*^*/mNos2*^*-/-*^ mice that have low levels of NO. However, *NOS2* is an inducible gene and any non-stimulated change in brain expression levels when the *HuNOS2* gene is inserted into the mouse genome is puzzling. *NOS2* translation and transcription are well-known to be regulated by disease-based immune activators including LPS and cytokines via multiple membrane receptors. For the human gene, *NOS2* gene expression is also regulated by non ‘cytokine’- like factors including β-catenin/Wnt2, epidermal growth factor (EGF), colony stimulating factor 1 (CSF1) and hormones such as testosterone [30, 38, 48]. Geller and colleagues [25, 30] have shown specific upstream sites in the human *NOS2* promoter that alter gene regulation. One of these sites mediates basal promoter activity of the *NOS2* gene and functions independently of known cytokine responsive regulatory elements in the gene promoter. The physiological role of basal induction of the *NOS2* gene in human cells is not well studied but has been implicated in changes that lead to cancer [48, 49]. To determine if non-stimulated “basal” changes could be found in mice expressing the human *NOS2* gene we compared mRNA levels of selected genes between saline- treated *mNos2*^*-/-*^ (where NO is not produced) and *HuNOS2*^*tg*^*/mNos2*^*-/-*^ mice that express a human *NOS2* gene and promoter and produce human levels of NO. Of the genes tested (Figure 4), Hsp27 was found to demonstrate a significant change in saline-treated (control) levels of mRNA expression with no significant difference in LPS-stimulated mRNA expression.

### Immune gene profiles are altered in mice expressing the *huNOS2*gene

**Figure 5:**
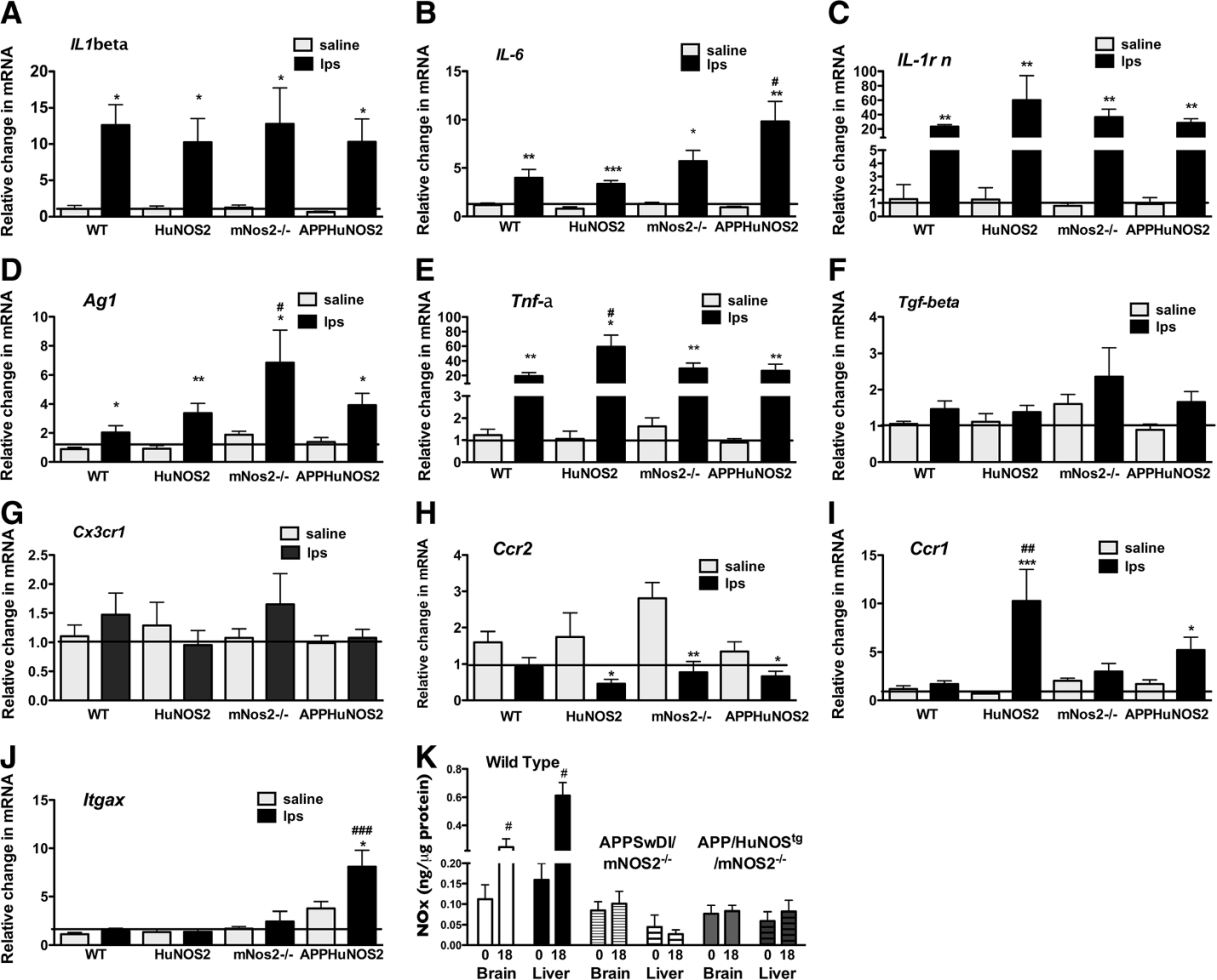
**Changes in immune gene expression levels in LPS-simulated** ***HuNOS2*** ^***tg***^ ***/mNos2*** ^***-/-***^ ***; APPSwDI*** ^***+/-***^ ***/Hu NOS2*** ^***tg+/+***^ ***/mNos2*** ^***-/-***^ **;** ***mNos2*** ^***-/-***^ **, and WT mice.** Brain lysates were prepared from saline-injected (gray bars) and LPS-injected (black bars) mice from each of the 4 different strains at 24 hrs. Saline-injected mice from each strain served as the corresponding 0 hrs time point. Average fold changes (±sem) for each gene were determined using the average WT saline-injected value as comparator. n = 5–6 mice per group. Panels **A**-**F**- cytokine genes; Panels **G**-**I**- chemokine genes - **J**- gene marker of activated microglia- integrin alpha10 (*CD11c*). Statistical significance was determined using two-way ANOVA with genotype as the between strain factor and treatment as the within strain factor (GraphPad Software, San Diego CA). Significance was set at p ≤0.05. *p <0.05; **p < 0.01; ***p <0.001; #p <0.05; ##p <0.01; ###p <0.001. **K**- APP mutations that increase Aβ production and amyloid deposition do not increase brain NOx levels. Brain lysates were prepared from saline injected and LPS injected *APPSwDI/mNos2* ^*-/-*^ and *APPSwDI* ^*+/-*^ */Hu NOS2* ^*tg+/+*^ */mNos2* ^*-/-*^ mice (see methods for details on mouse strains). NOx brain lysate levels in LPS-stimulated WT mice serve as the positive control for NO production. Data are presented as the average NOx values ± sem (n = 4–6 mice per group). Significant differences were determined for each tissue type separately using two-way ANOVA with genotype as the between strain factor and treatment as the within strain factor. #p <0.05.

We next determined if changes in the tissue redox responses in *HuNOS2*^*tg*^*/mNos2*^*-/-*^ mice could also impact cytokine production in the brain. Thus, gene expression levels for specific chemokines, pro-inflammatory and anti-inflammatory cytokines were measured in *HuNOS2*^*tg*^*/mNos2*^*-/-*^ mice and compared to WT and *mNos2*^*-/-*^ mice at 24 hrs after LPS injection. We also measured the same genes in brain lysates from the *HuNOS2*^*tg*^*/APPSwDI/mNos2*^*-/-*^ to understand if brain Aβ production and accumulation altered cytokine mRNA levels when the *HuNOS2* gene is expressed. All mice were injected with 10 mg/kg LPS or saline and brain lysates prepared in the same manner as described previously. Gene expression levels are presented in Figure 5 as the average (±sem) fold changes observed under untreated and LPS- treated conditions. As shown, pro-inflammatory (*Il-1β; TNFα, IL-6*) and anti-inflammatory (*Ag1*, *IL-rn*) mRNA levels significantly increased with LPS- treatment in each of the mouse strains examined (Figure 5 A-E). Genotype specific differences were also observed. Significantly greater responses were observed in APPhuNOS2 mice for *Il-6*, in *mNos2*^*-/-*^ mice for *Ag1* and in *HuNOS2*^*tg*^*/mNos2*^*-/-*^ mice for *Tnfα. Tgfβ* failed to show a response to LPS in any of the mouse strains (Figure 5F). Chemokine gene expression patterns were dependent on the specific chemokine. *Cx3cr1* mRNA expression (Figure 5G) was not significantly altered by treatment with LPS in any of the mouse strains. For *Ccr2,* LPS- treatment reduced levels of mRNA expression in brain lysates from *HuNOS2*^*tg*^*/mNos2*^*-/-*^, APPHuNOS2 and *mNos2*^*-/-*^ mice but not in WT mice (Figure 5H*)*. However, increased LPS-mediated expression of *Ccr1* was found only in the strains that expressed *HuNOS2*^*tg*^*/mNos2*^-/-^. We also examined a microglia marker that is found in patients with AD [9, 10]. The *Itgax* gene encodes the CD11c protein and was significantly increased only in *HuNOS2*^*tg*^*/APPSwDI/mNos2*^*-/-*^ mice (Figure 5J). Finally, to determine if the production of Aβ peptides and accumulation of amyloid altered nitrate and nitrite levels in brain, we measured NOx levels as described previously in brain and liver lysates from *HuNOS2*^*tg*^*/APPSwDI/mNos2*^*-/-*^ and *APPSwDI/mNos2*^*-/-*^ mice using WT as the positive control. No significant changes in NOx levels were observed for mice from either of the APP strains (Figure 5K).

**Figure 6:**
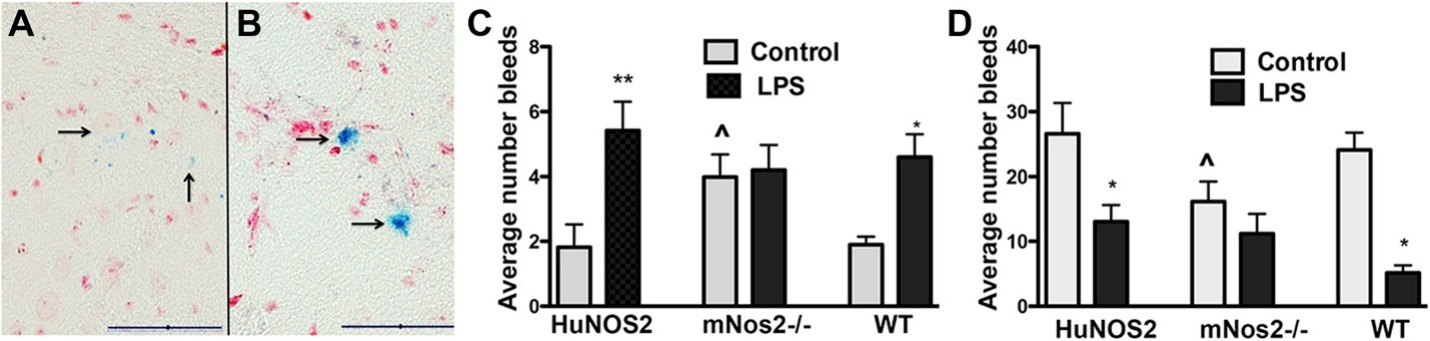
**Mouse stain specific differences in brain microhemorrhage.** The total number of microhemorrhages per brain section were calculated for *HuNOS2* ^*tg+/+*^ */mNos2* ^*-/-*^; *mNos2* ^*-/-*^, and WT mice under saline treated (control-gray bars) and LPS-treated (black bars) conditions. Panel **A**; **B**- typical view of small (<20 microns; **A**) and large (>20 microns; **B**) microbleeds observed in brain sections using Prussian blue histochemistry to identify hemisedrin deposits. Panel **C**; **D**- Data represent the average number (+ sem) of microbleeds/section (n- 4-7 mice of each strain) for control and LPS-treated mice. Significant differences were determined using two-way ANOVA with genotype as the between strain factor (^p<0.05) and treatment as the within strain factor (#p < 0.05; ##p<0.01).

The observed changes in brain gene expression between WT, *HuNOS2*^*tg*^*/mNos2*^*-/-*^ and *mNos2*^*-/-*^ mice suggested that strain-dependent physiological differences may also be found. LPS treatment is well known to affect the brain /blood interface by increasing small hemorrhages resulting in brain accumulation of red blood cells and hemosiderin, a break down product of heme iron [50–52]. Since the blood brain barrier integrity is regulated, in part, by NO-dependent metalloprotease (MMP) activity [52, 53] we compared the average number of brain microbleeds per section (±sem) in WT, *HuNOS2*^*tg*^*/mNos2*^*-/-*^ and *mNos2*^*-/-*^ mice. Microbleeds were assessed using Prussian blue histochemistry as previously described [51, 52] and categorized as “small” (Figure 6A) or “large” (Figure 6B). As shown in Figure 6C, LPS-treatment initiated a significant increase in large microbleeds compared to saline-treated (control) brains in both *HuNOS2*^*tg*^*/mNos2*^*-/-*^ and *mNos2*^*-/-*^ mice. Mice lacking *Nos2*, however, showed a significant increase in untreated control brain bleeds when compared to untreated *HuNOS2*^*tg*^*/mNos2*^*-/-*^ and *mNos2*^*-/-*^ brains. The average untreated value from *mNos2*^*-/-*^ brains was not different compared to LPS-treated *mNos2*^*-/-*^ brains. A similar pattern was observed for small bleeds in terms of the strain differences. However, the number of small bleeds was significantly lower with LPS treatment (Figure 6D).

## Discussion

NO and superoxide anion are primary redox molecules that establish and regulate tissue redox balance through direct and indirect mechanisms of action. Both NO and superoxide anion are also central components of immunity with regulatory and defensive roles. Thus, it is likely that species differences in production or consumption of either NO or superoxide anion will have evolutionary significance. As a reactive molecule that interacts with multiple proteins and has a relatively long diffusion distance, NO is particularly well suited to impact cellular pathways. The outcome of these reactions is now well known to be dependent on the level of NO [34, 42, 48, 54, 55]. Thus, understanding how and when NO levels change in a tissue is not only critical to deciphering redox regulated mechanisms but also is critical to unraveling immune processes and immune pathology.

The discovery of miRNA-939 mediated regulation of human iNOS protein translation by Guo et al. [29] has provided a realistic mechanism to explain a long term conundrum: Why is iNOS mediated production of NO different between human and rodent? Our data support their finding and further directly show that these differences in NO production impact the redox, immune and physiological signature of the brain. This was accomplished by reconstituting human *NOS2* into a mouse *Nos2* knockout model, thus providing a useful tool to study NOS2/NO related responses that are relevant to human disease in an *in vivo* as well as *in vitro* setting. Our data from *HuNOS2*^*tg*^*/mNos2*^*-/-*^ mice clearly show the presence of the binding site for miRNA-939 in the 3′UTR region of the *HuNOS2* gene and the presence of a miRNA-939 homolog in the brain, thus recreating a more human-like condition. While C57Bl/6 mice express miRNA-939, they lack the binding site in the gene and the additional regulatory complexity found in the human *NOS2* gene promoter. Geller and colleagues [25, 30] have also described unique promoter sites in the human gene that involve non-cytokine based regulatory elements and elicit basal production of NO by NOS2. This difference in NO regulation implies a more varied and complex production of NO in human tissues.

The inability to readily measure nitrite and nitrate levels as a surrogate marker of NO production in human immune cells has provided an increased level of difficulty in charting NO’s involvement in specific pathways. As shown in this study, LPS-mediated stimulation of human NOS2 *in vivo* does not result in measureable increases in brain lysate levels of nitrite and nitrate, the commonly used indicators of NO production. Nitrate and nitrite are clearly not the only reaction product of NO, however, and other reactive nitrogen species are produced as a result of human NOS2 activation during an immune response [39–41]. The directed gene array for *HuNOS2*^*tg*^*/mNos2*^*-/-*^ mice was designed to provide additional insight into the spectrum of pathways that may be differentially activated by human *NOS2* vs mouse *Nos2*. We specifically chose redox based genes to profile because of NO’s inherent redox activity and the role of redox-based regulation in inflammation. In addition, neurodegenerative diseases of aging such as Alzheimer’s disease have oxidative stress as a primary component [56–58]. A similar directed array approach has recently been used to delineate redox genes that are changed in humans with Multiple Sclerosis [59].

Overall, our array results show that LPS induction in mice expressing reconstituted *huNOS2* produced a statistically significantly different pattern of redox gene expression than WT mice. The exact inter-relationships between NOS2 expression levels and specific gene expression levels such as cystathione b synthase (*Cbs*) and glutathione peroxidase 3 (*Gpx3*) are not precisely defined here. For some genes such as *Cbs*, however, direct connections to NO are known. For example, the enzymatic activity of Cbs protein is reduced when NO binds to the heme site of the protein, thereby altering the transulfuration pathway and the production of either hydrogen sulfide (H2S) or glutathione (GSH) [60]. These data coupled with the increased expression of glutathione peroxidase-3 and glutathione reductase in only LPS-treated WT mice imply a regulatory cross talk between glutathione, H_2_S and high levels of NO which may not be observed in either the *mNos2*^*-/-*^ or *HuNOS2*^*tg*^*/mNos2*^*-/-*^ strains where NO generated via NOS2 is low.

Two other important and specific points can be gleaned from our data set. First, re-constitution of the human *NOS2* gene restores gene expression changes that occur as a result of *mNos2* depletion. For example, genes such as *Lpo* and *Ag1* are elevated only in *mNos*^*-/-*^ mice. In mice expressing human *NOS2* mRNA, both *Lpo* and *Ag1* expression levels are restored to the WT equivalent. These data support the functionality of the HuNOS2 gene in the *mNos*^*-/-*^ mouse background. Secondly, the human *NOS2* gene shows unique characteristics that are not found in either WT or mice lacking *mNos2*. For example, three key genes associated with disease, that is, heme-oxygenase 1 (*Ho1*), *TNFα* and *Ccr1* are upregulated in *HuNOS2*^*tg*^*/mNos2*^*-/-*^ mice brain compared to either WT or *mNos*^*-/-*^ mice. These changes are likely to impact both the response to hypoxia and the response of the brain to immune challenge. The presence of human *NOS2* also mitigates the apparently “leaky” cerebral vessels found in *mNos2*^*-/-*^ mice. Increased constitutive levels of both large and small microglia bleeds were observed in knock-out mice. Although LPS altered the number of hemorrhage sites in WT and *HuNOS2*^*tg*^*/mNos2*^*-/-*^ mice in a complex manner, addition of the human *NOS2* gene prevented these changes under normal, non-LPS simulated levels, thus mimicking WT mice.

Differences in NO production and its complex action in the brain’s redox environment clearly contribute to the frequently conflicting data and disparate views of NO’s role in disease, particularly when mouse models are used. For neurodegenerative disease, NO is commonly viewed as a contributor to neuronal death in the brain through its ability to interact with superoxide ion to form reactive oxygen species that kill the cell [61, 62]. A good example of this is the expression of iNOS in neurons in APP mouse models of AD and in patients with AD. Cortical neurons from the APPSw (Tg2576) mouse model of AD show iNOS immunoreactivity around amyloid deposits and in neurons associated with intracellular Aβ [63]. While footprints of oxidation are found in this micro-environment, the primary event that generates reactive oxygen species may actually be the loss of NO’s role as a superoxide scavenger to reduce oxidative stress [42, 64]. Local oxidants such as reactive iron can interact with NO, leading to the formation of nitrotyrosine and reducing local NO levels [65]. In mouse brain where NO levels are likely to be high, the impact of the loss of NO in the environment will be different than in human brain where the inherent levels of NO generated by iNOS are likely to be restricted by the complexity of the *NOS2* gene promoter and by miRNA regulation of translation. Rodrigo et al. [63] clearly show that increased expression of iNOS in APPSw mice is accompanied by increased Aβ and oxidative outcomes, but these are not accompanied by neuronal death. *APPSw* Tg2576 mice do not show neuronal loss [66]. In contrast, the removal of *mNos2* as shown in the *APPSw/NOS2*^*-/-*^ mice and the *APPSwDI/NOS2*^*-/-*^ mice recreate a NO –poor micro-environment and do demonstrate neuronal death. We also have recently shown that reconstitution of human *NOS2* into mice expressing mutated human APP and that lack *mNos2* also proceed to neuronal loss [67], further suggesting a critical role of NO levels. Fernandez-Vizarra [68] has carefully examined the expression of nNOS and iNOS at different stages of AD in neurons from humans with AD. Interestingly, their data demonstrated an increasing level of expression of nNOS followed by increasing ecotopic expression of iNOS in cortical neurons. They also observed that the increased expression of NOS enzymes with progression of AD pathology was frequently accompanied by nitrotyrosine immunoreactivity but was only rarely associated with signs of neuronal death. They concluded that the presence of NO was protective until “oxidative” events became a dominant cellular pathology. The inherently different regulation of NO levels produced by human iNOS during immune activation as we show herein and/or the consumption of arginine, the sole substrate of iNOS are likely to be additional factors that lead to the worsening pathology in humans with AD. Thus the levels of NO and its perspective chemical biology in the microenvironment is an important determinant of outcome.

Mouse genetic models are, and undoubtedly will remain, a significant tool to study neurodegeneration and neurological disease. The importance of their contribution to our understanding of the disease process in turn depends on how the genetic change enables production of a pathological pathway that resembles the pathway found in humans. The importance of this question ultimately depends on the degree of association of the gene changes with disease. As discussed previously, Zhang’s [17] data derived from gene networks analysis of autopsied brain samples from humans with AD provide important insights. Their studies clearly show a dominant contribution of microglial/immune genes to multiple clinical co-variants found in patients with AD. However, NO mediated network associations with AD pathology are minimal. It is likely, then, that consideration of differences between human and mouse *NOS2* and the impact this has on the brain’s redox environment may be useful in studying human disease including AD.

## Methods

### Animals

All animal experiments were performed in accordance with protocols approved by the Institutional Animal Care and Use committee at Duke University Medical Center under the NIH Guide for the Utilization and Care of Vertebrate Animals Used in Testing, Research and Training.

### *Mice strains: HuNOS2*^*tg+/+*^*/mNos2*^*-/-*^mice

The *HuNOS2*^*tg+/+*^*/mNos2*^*-/-*^ strain was developed and characterized by Vitek et al. as described [45].

### *APPSwDI*^*+/-*^*/Hu NOS2*^*tg+/+*^*/mNos2*^*-/-*^mice

Homozygous *APPSwDI/mNos2*^*-/-*^ (CerebroVascular amyloid-*Nos*2^-/-^ or CVN-AD) mice were produced by crossing mice expressing the vasculotropic Swedish K760N/M671L, Dutch E693Q and Iowa D694N human *APP* mutations under control of the Thy-1 promoter with *mNos2*^*-/-*^ (B6 129P2NOS2^tau1Lau^/J) mice [45]. These mice were then crossed to *HuNOS2*^*tg+/+*^*/mNos2*^*-/-*^ mice to generate the *APPSwDI*^*+/-*^*/Hu NOS2*^*tg+/+*^*/mNos2*^*-/-*^ strain.

### Control mice

*mNos2*^*-/-*^ (B6 129P2NOS2^tau1Lau^/J) and C57Bl/6 WT mice were purchased from Jackson Laboratory, Bar Harbor ME and produced through the barrier breeding colonies at Duke University. All mice were genotyped in a standard fashion.

### LPS treatment

Mice between the ages of 36–40 weeks of age (mixed genders) were *iv* injected with 10 mg/kg LPS or saline (0.9% NaCl) via the tail vein. Mixed genders were used in the analyses and gender based-differences were not investigated. Mice were allowed to recover on heated pads and then were humanely euthanized at 7 and 24 hrs after the injection with a lethal mixture of ketamine/xylazine. Each mouse was intracardially perfused with approximately 25 mls of phosphate buffered saline (PBS). Hemispheres from perfused brains and 2–3 lobes from each liver were then rapidly removed, immediately frozen in liquid nitrogen and the cryo-preserved tissue was pulverized under liquid nitrogen for use in the assays. The remaining hemispheres were fixed in 4% paraformaldehyde and passed through sucrose gradients for sectioning on a freezing microtome.

### Prussian blue staining

Sagittal sections (25 microns) were cut from fixed brain, mounted and air-dried on slides for staining using the Perls protocol. After rehydration, sections were reacted with acidic potassium ferrocynanide (2 gm/100 mls) for 30 mins. Sections were then counterstained with neutral red. The number of microbleeds was counted at 20 × magnification for 4 complete brain sections/mouse located between 0.6-2.04 mm lateral to the bregma for a minimum of 4 mice per strain. Microbleeds were identified as bright blue spots and were separated into two categories; large (>20 microns) and small (<20 microns) (see Figure 6).

### NOx assay

For the NOx assay 40 mg of frozen pulverized brain or liver tissue from each brain were boiled in 250 μl PBS buffer for 10 min. After centrifugation to pellet, total nitrite and nitrate (NOx) content of the supernatants were measured using a Sievers Nitric Oxide Analyzer under reduction by vanadium (III) chloride (VCl_3_) in 1 N HCl, heated to 95°C [69]. NOx levels were normalized to total protein content of the supernatants as determined by BCA assays.

### qRT-PCR

Total RNA was extracted from approximately 40 mg of frozen pulverized brain tissue, which was homogenized using a Bullet Blender with RNase free 0.5 mm zirconium oxide beads (Next Advance) in TRIzol reagent (Life Technologies). cDNA was produced using the cDNA High Capacity kit (Life Technologies) according to the manufacturer’s instructions. Real-time PCR was performed using TaqMan Gene Expression assays (Life Technologies) also according to the manufacturer’s instructions and as previously described [70]. Data were normalized to either 18 s or ß-actin. Specific miRNA was reverse transcribed using the TaqMan MicroRNA Reverse Transcription Kit (Life Technologies) and the specific RT primer supplied with each TaqMan miRNA assay. Quantitative PCR was then run using the BioRad CFX96 Touch Real Time PCR detection system. Average fold change values (RQ) were determined by 2^(-ΔΔCt)^ method using saline injection as the comparator for individual strain samples [47].

*Primers:* Primers for WT *mNos2* were: Forward 5′-TTA CGT CCA TCG TGG ACA GC-3′and for Reverse 5′-TGG GCT GGG TGT TAG TCT TA-3′. Primers for the human *3′UTR* were: Forward- 5′ CCC CCA GCC TCA AGT CTT ATT TC-3′ and for Reverse- 5′-CAG CAG CAA GTT CCA TCT TTC AC-3′. Primers for dectection of miRNA-939 were purchased as a TaqMan MicroRNA assay (Assay name- hsa-mir-939) from Life Technologies.

### Statistical analysis

Data are presented as average values ± SEM. Where appropriate, significant changes within strain were determined using student’s *t*-test or one-way ANOVA while statistical significance between strains and treatments was determined using two-way ANOVA (GraphPad Software, San Diego CA). Significance was set at p ≤0.05.
